# Outcomes of patients with anal cancer treated with definitive chemoradiation: A single centre experience

**DOI:** 10.3332/ecancer.2024.1655

**Published:** 2024-01-15

**Authors:** Mirza Rameez Samar, Bakhtawar Masood, Nida E Zehra, Tahir Munir, Misbah Younus Soomro, Muhammad Arif Hameed, Insia Ali, Yasmin Abdul Rashid

**Affiliations:** 1 Department of Oncology, The Aga Khan University, Karachi 74600, Pakistan; 2 Department of Anaesthesia, The Aga Khan University, Karachi 74600, Pakistan

**Keywords:** anal cancer, definitive chemoradiation, squamous cell carcinoma, non-metastatic, disease-free survival

## Abstract

**Background:**

Anal cancers are uncommon neoplasms that make up to <1% of all tumours globally. Concurrent chemoradiation remains the standard of care treatment for patients who present with non-metastatic anal squamous cell carcinomas (ASCCs).

**Methods:**

We aimed to evaluate the response rate and 2-year survival outcome of the definitive chemoradiation approach in patients with non-metastatic ASCCs of our population. We conducted a cross-sectional review of these patient populations who were treated and then followed after completion of treatment at our institute during the last 10 years.

**Results:**

A total of 17 patients were enrolled after fulfillment of the eligibility criteria. The responses were documented in 16 patients through magnetic resonance imaging or computed tomography of the pelvis, done at 3 months of treatment completion. More than 80% of the patients had complete radiological responses. Among the surviving participants, the 2-year disease-free survival rate was found to be more than two-thirds. Approximately 20% of the study participants had disease recurrence during the subsequent clinic visits following treatment completion.

**Conclusion:**

This review emphasises the impact of definitive chemo-radiation in achieving radiological and clinical responses in patients with non-metastatic ASCCs. Moreover, to our knowledge, this is the first review to highlight anal cancer’s incidence and characteristics in Pakistan.

## Background

Neoplasms arising from the anal canal are referred to as anal cancers. They comprise <1% of all cancer cases diagnosed in the United States. In Pakistan, they give rise to approximately 0.23% of all the cancers diagnosed in our country [1]. Among the various subtypes, anal squamous cell carcinoma (ASCC) is the predominant subtype, constituting up to >80% of all anal cancer cases. The state-of-the-art treatment of non-metastatic ASCC is a non-surgical, multimodal approach of chemo-radiotherapy (CRT) i.e., radiotherapy given with chemotherapeutic agents as radio-sensitisers. The current CRT approach involves 5-fluorouracil (5FU) and mitomycin C with radiotherapy, which provides a local disease control rate of 59% at 42 months when compared to 36% with radiotherapy alone [2].

In this study, we aim to determine the response rates, 2-year disease-free survival (DFS), and patterns of recurrence from a combined CRT approach in patients with non-metastatic anal cancer presenting at a tertiary care institute in Pakistan.

## Methods

We conducted a retrospective review involving patients with non-metastatic ASCC from 2011 to 2020, at the Department of Oncology, The Aga Khan University Hospital, Karachi. The patients were enrolled regardless of their tumour size and nodal involvement. The tumour was designated as per the tumour, node and metastasis (TNM) staging system (9th ed., 2022). All the patients received and completed their treatment at The Aga Khan University Hospital. Those patients with non-squamous histology were excluded, and so were the patients with recurrent or metastatic anal cancer as well as those patients who were treated outside the institute. The confidentiality of the patients was maintained by assigning a predefined serial number instead of using the medical record numbers. The response was documented by contrast-enhanced magnetic resonance imaging (MRI) or computed tomography (CT) of the abdomen and pelvis done at 12 weeks of completion of CRT. The response was categorised as complete response (CR), partial response (PR), no response (NR), or progressive disease (PD). CR was defined as the radiographic eradication of all identifiable tumour. PR was defined as a radiographic disease reduction by at least 30% or more. NR was defined as no radiographic change in tumour extent. Recurrent disease was documented by the presence of disease on contrast-enhanced MRI of the abdomen and pelvis ± contrast-enhanced CT of the chest, repeated three monthly in the first 2 years and then six monthly in the third year of surveillance. The clinical data was obtained from electronic or paper records. The study was conducted following approval from the institutional review board of The Aga Khan University Hospital.

The data was entered and analysed using Statistical Package for Social Science version 19 (Chicago, IL, USA). Categorical variables were analysed as frequency, percentage, and graphical representations. To assess the normality assumption of numerical variables, either the Shapiro–Wilk test or the Kolmogorov-Smirnov test was employed. The results indicated that the data did not follow a normal distribution. Consequently, median estimates (minimum and maximum values) were computed.

## Results

### Baseline demographics and clinical characteristics

A total of 17 patients were enrolled in the study after fulfilling the inclusion criteria. The median age of the patients was 61 years. Males were approximately twice in number to that of females. Eight patients (47.1%) did not have a co-existing illness and hypertension was the most frequent co-morbid illness (29.4%). The HIV status of less than half of the participants was known, all of whom were negative. Eight patients (47.1%) had moderately differentiated tumours. T3 and T4 tumour sizes were evenly distributed, with each being found in five patients (29.4%). Nine patients (52.9%) had N1 nodal status, with N1a found in more than half of the patients with nodal involvement. Most of the patients (64.7%) diagnosed belonged to stage III (Table 1).

### Treatment

Of the 17, 13 (76.5%) patients received 5’FU-based chemoradiation. 12 (92.3%) of these 13 patients received 5’FU at 1,000 mg/m**^2^** from day 1 to 4 and from day 29 to 32. All of the four remaining participants received per-oral capecitabine at 825 mg/m**^2^** every 5 out of 7 days. The median dose of mitomycin received was 10 mg/m**^2^**, administered on day 1 of concurrent CRT. The majority (82.4%) of the participants received 5,940 Gray (Gy) of radiotherapy, divided into 33 fractions (Fr). The median duration of the entire CRT period was 6.5 weeks. All of the patients had drug-related acute toxicities, most of which were of grade 1. The most frequent adverse event was radiotherapy-induced skin reaction, seen in all of the participants. This was followed by myelosuppression (predominantly leukopenia and thrombocytopenia), seen in 10 (58.8%) participants. Other acute adverse events of significance included nausea and vomiting (35%), diarrhoea (23.5%), alopecia (23.5%), anorexia (17.6%) and stomatitis (17.6%). Only 2 (11.8%) of 17 patients had treatment interruptions due to infection. None of the patients had drug-related toxicity that led to treatment interruption (Table 2).

### Response rates

**Figure 1.**
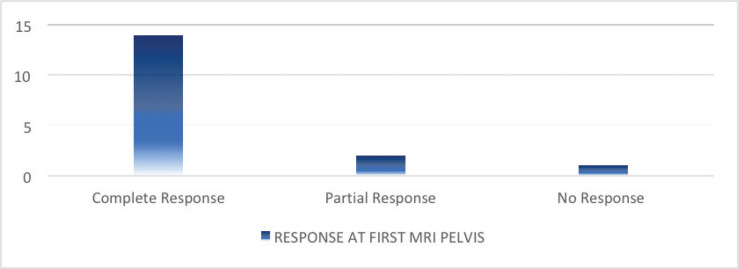
Response to CRT at first MRI pelvis.

Among the 17 participants with ASCC, 83.5% of the patients had a complete radiological response on the follow-up imaging performed at 12 weeks after the completion of definitive chemoradiation (Figure 1).

### Recurrence and pattern of recurrence

**Figure 2.**
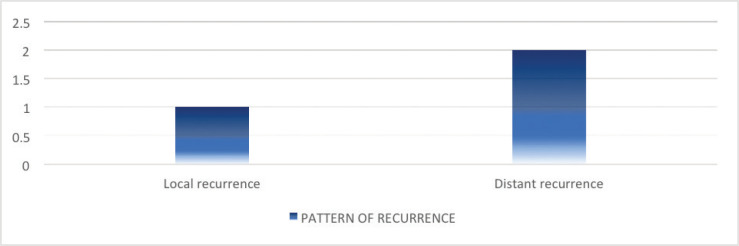
Pattern of recurrence following definitive chemoradiation.

Of the 16 patients with radiologically responsive disease, cancer recurrence was documented in 3 (21.4%) patients on subsequent imaging upon follow-ups. Among these, distant recurrence was found in 2 (33.3%) patients. Of these, only one patient received systemic therapy upon recurrence (Figure 2).

### Disease-free survival

Upon subsequent visits, 15 of 17 (88.4%) patients with ASCC were found to be alive at 24 months of definitive treatment. In a subgroup analysis (Table 3), approximately 93% of the participants with CR at first MRI pelvis, were found to be alive at 2 years, which was statistically significant (*p* = <0.001).

## Discussion

Anal cancers are commonly found in the United States with an estimated worldwide incidence of 50,865 new cases diagnosed each year and a yearly mortality rate of 19,293 cases [3]. They do not have age preponderance or gender predisposition. Histologically, these can be divided into various subtypes including squamous cell carcinomas, adenocarcinomas, adeno-squamous carcinomas, small cell carcinomas, melanomas and carcinoids. Over the past 30 years, there has been a three-fold rise in the incidence of ASCC, mainly in women [4]. Around 90% of such cases can be attributed to human papillomavirus (HPV). A wide variety of strains are known to carry the risk of ASCC including HPV6/11/16/18/31/33/45/52/58. Among these, HPV 16 and 18 are the high-risk genotypes, contributing to >85% of ASCC [5]. Patients harbouring these genotypes are at risk for premalignant lesions known as high-grade anal intraepithelial neoplasia i.e., grade 2 and 3. Other risk factors include the increased prevalence of receptive anal sex, prior history of cancer, anogenital dysplasia, immunosuppression (in HIV/AIDS patients or transplant recipients) and less commonly tobacco use [6].

During the early 1970s, the primary treatment of non-metastatic ASCC was abdominoperineal resection (APR), which despite yielding a 5-year survival rate between 40% and 70% [7] was associated with significant morbidity, due to the lack of sphincter preservation and the presence of permanent colostomy. This resulted in a transition over time in state-of-the-art treatment to a multimodal non-surgical approach that involves concurrent chemotherapy and radiotherapy (CRT). Various studies have shown a CR rate of more than 80% with the CRT approach [8–10]. APR is now usually reserved for patients who failed standard treatment or at the time of locoregional relapse as salvage therapy. Even at a 13-year follow-up, the combined CRT modality demonstrated superiority in local disease control, colostomy-free survival and disease-specific survival [11]. In a retrospective review conducted by Kumar *et al* [12], definitive CRT yielded a 3-year DFS of 73.9% in patients with non-metastatic ASCC. Most of these participants had stage III disease and moderate histological differentiation, which parallels the traits of the patients enrolled in our study [12].

The prognosis of ASCC is largely influenced by multiple factors including nodal involvement, tumour size, skin ulceration, gender and HPV. Yuan *et al* [13] reported the differences in 5-year survival rates between node-negative and node-positive disease (76.7% versus 70.3%), and between stages I–II and stages III–IV (83.6% versus 71.2%), respectively. Esin *et al* [14] also reported an improvement in progression-free survival (PFS) with a lower T stage [14]. In addition, immunochemical staining of p16 also has prognostic value, with p16-positive tumours having a 2-year relapse-free rate of 78.2%, compared to 49.0% in p16-negative tumours, as reported by Wakeham *et al* [15].

In our study, we looked at the treatment outcomes of the definitive CRT approach in terms of first radiological response on CT or MRI pelvis post-completion of CRT and also DFS in patients with non-metastatic ASCC, treated in a tertiary care hospital, in Karachi, Pakistan. In our study, more than two-thirds of the participants had complete radiological response following CRT, which is consistent with the responses seen in previous studies [16–20]. In a Turkish study evaluating HIV-negative patients, 100% response rate was demonstrated with the CRT approach in patients with ASCC, all of whom were CRs [14]. A similar study of 48 patients with ASCC was conducted by Day *et al* [21] in which CRs were documented in 79% of the patients via F-fluorodeoxyglucose positron emission tomography [21].

Despite most of the patients receiving 5’FU-based chemoradiation, the response rates did not differ from those who received capecitabine during CRT. Our study demonstrates a 2-year DFS of >80% which is comparable to other studies [15, 20–26]. The 2-year DFS was significantly associated with the response at first MRI pelvis. The recurrence rate was low in our analysis and distant recurrence was more than local recurrence.

Our study also had its limitations. This is a retrospective, single-centre study. Prospective trials should be designed in the future to control additional factors associated with treatment outcomes. The small sample size would have impacted the recurrence rate, this could be depicted largely in part, due to the low overall incidence of ASCC worldwide. Also, the known number of recurrences in this study is too small to reflect the actual impact of the stage, nodal status and to make any inference regarding the pattern of recurrence after completion of CRT. Moreover, the immunohistochemical status of p16 was not certified in our participants, which is documented to play an integral role in determining the prognosis of such cases.

## Conclusion

This review highlights the significance of CRT approach in the management of ASCC. Being an original report from Pakistan, it serves as a footing for continued investigation into the landscape of anal cancers within our territory. Moreover, with the promotion of awareness, prompt diagnosis can be encouraged and proper management can be employed, which may facilitate in diminishing the existing load of anal cancers in our vicinity.

## Ethics approval and consent to participate

This study was approved by Institutional Ethics Review Committee (ERC number: 2022-7963-22909) and all participants provided written informed consent.

## Competing interests

The authors declare that they have no competing interests.

## Funding

No specific funding has been used for manuscript writing or reporting.

## Consent for publication

Not applicable.

## Availability of data and materials

The data that support the findings of this study are available from the corresponding author upon reasonable request.

## Author contributions

MRS, BM and YAR were instrumental in shaping the initial manuscript. Notably, MRS took a central role in conceptualising, refining format and enhancing the final rendition. MS and IA earnestly participated in reviewing and providing crucial input. YAR and MAH meticulously examined the concluding draft. All authors unanimously endorsed the final version for publication. NZ and TM adeptly conducted data analysis, contributing their statistical expertise. Furthermore, NZ played a key role in the final manuscript submission process.

## Figures and Tables

**Table 1.** Demographic and baseline characteristics of the study population.

Variable	Overall (*n* = 17)
Age	
Mean (SD)	62.0 (±11.9)
Median (Min, Max)	61.0 (40.0, 79.0)
Gender	
Female	6 (35.3%)
Male	11 (64.7%)
ECOG status	
1	10 (58.8%)
2	6 (35.3%)
3	1 (5.9%)
Comorbid illness	
CVA	1 (5.9%)
DM	1 (5.9%)
HTN	5 (29.4%)
IHD	2 (11.8%)
None	8 (47.1%)
HIV status	
Positive	0 (0%)
Negative	7 (41.2%)
Unknown	10 (58.8%)
Histological differentiation	
Moderately differentiated	8 (47.1%)
Poorly differentiated	5 (29.4%)
Well-differentiated	4 (23.5%)
Pre-treatment clinical tumour size	
T1	3 (17.6%)
T2	4 (23.5%)
T3	5 (29.4%)
T4	5 (29.4%)
Pre-treatment nodal involvement	
N0	8 (47.1%)
N1a	7 (41.1%)
N1b	1 (5.9%)
N1c	1 (5.9%)
Pre-treatment clinical stage	
Stage I	3 (17.6%)
Stage II	3 (17.6%)
Stage III	11 (64.7%)

**Table 2.** Treatment details of definitive chemoradiation.

Variable	Overall (*n* = 17)
Chemotherapy regimen used for CCRT	
Mitomycin + 5’FU	13 (76.5%)
Mitomycin + capecitabine	4 (23.5%)
Dose of 5’FU (*n* = 13)	
1,000 mg/m^2^	12 (70.6%)
750 mg/m^2^	1 (5.9%)
Dose of capecitabine (*n* = 4)	
825 mg/m^2^	4 (23.5%)
Dose of mitomycin	
10 mg/m^2^	13 (76.5%)
15 mg/m^2^	3 (17.6%)
8 mg/m^2^	1 (5.9%)
Dose modification needed	
Yes	1 (5.9%)
No	16 (94.1%)
Fractions of radiotherapy (in number)	
28	2 (11.8%)
30	1 (5.9%)
33	14 (82.4%)
Total dose of radiotherapy (in Gy)	
5,040	2 (11.8%)
5,940	14 (82.4%)
6,000	1 (5.9%)
Technique of radiotherapy	
Intensity-modulated radiotherapy	10 (58.8%)
Three-dimensional conformal radiotherapy	7 (41.1%)
Duration of treatment (in weeks)	
Mean (SD)	6.62 (0.697)
Median (Min, Max)	6.50 (6.00, 9.00)
Any interruption in treatment	
Yes	2 (11.8%)
No	15 (88.2%)
Reason of interruption	
Infection	2
Toxicity	0
	


**Table 3.** Association of treatment response to 2-year survival outcome.

Response at first MRI pelvis post-completion of CRT	Patient alive at 2 years of treatment completion		Total patients (*n* = 17)	*p*-value
	No	Yes		
CR	1 (7.1%)	13 (92.9%)	14 (100%)	<0.001
NR	0 (0%)	1 (100%)	1 (100%)	0.159
PR	1 (50%)	1 (50%)	2 (100%)	>0.900

## References

[1] Sung H, Ferlay J, Siegel RL (2021). Global Cancer Statistics 2020: GLOBOCAN estimates of incidence and mortality worldwide for 36 cancers in 185 countries. CA Cancer J Clin.

[2] (1996). Epidermoid anal cancer: results from the UKCCCR randomised trial of radiotherapy alone versus radiotherapy, 5-fluorouracil, and mitomycin. UKCCCR Anal Cancer Trial Working Party. UK Co-ordinating Committee on Cancer Research. Lancet.

[3] Siegel RL, Miller KD, Fuchs HE (2022). Cancer statistics. CA Cancer J Clin.

[4] Fish R, Sanders C, Williamson PR (2017). Core outcome research measures in anal cancer (CORMAC): protocol for systematic review, qualitative interviews and Delphi survey to develop a core outcome set in anal cancer. BMJ Open.

[5] de Martel C, Plummer M, Vignat J (2017). Worldwide burden of cancer attributable to HPV by site, country and HPV type. Int J Cancer.

[6] Urbute A, Munk C, Sand FL (2022). Trends in incidence and survival from anal cancer and incidence of high-grade anal intraepithelial neoplasia in Denmark. Cancer Epidemiol.

[7] Dee EC, Byrne JD, Wo JY (2021). Evolution of the role of radiotherapy for anal cancer. Cancers (Basel).

[8] Bartelink H, Roelofsen F, Eschwege F (1997). Concomitant radiotherapy and chemotherapy is superior to radiotherapy alone in the treatment of locally advanced anal cancer: results of a phase III randomized trial of the European Organization for Research and Treatment of Cancer Radiotherapy and Gastrointestinal Cooperative Groups. J Clin Oncol.

[9] Doci R, Zucali R, La Monica G (1996). Primary chemoradiation therapy with fluorouracil and cisplatin for cancer of the anus: results in 35 consecutive patients. J Clin Oncol.

[10] Peiffert D, Seitz JF, Rougier P (1997). Preliminary results of a phase II study of high-dose radiation therapy and neoadjuvant plus concomitant 5-fluorouracil with CDDP chemotherapy for patients with anal canal cancer: a French cooperative study. Ann Oncol.

[11] Northover J, Glynne-Jones R, Sebag-Montefiore D (2010). Chemoradiation for the treatment of epidermoid anal cancer: 13-year follow-up of the first randomised UKCCCR Anal Cancer Trial (ACT I). Br J Cancer.

[12] Kumar V, Bansal S, Badola A (2022). Definitive chemoradiation in nonmetastatic squamous cell carcinoma anal canal: a single-institution experience. J Cancer Res Ther.

[13] Yuan Y, Xie WH, Li RZ (2022). Comprehensive treatment experience of anal squamous cell carcinoma from a tertiary cancer center in South China. Cancer Med.

[14] Esin E, Yıldız F (2018). Real world survival data of a rare malignancy: Anal cancer results in HIV negative patients from Turkey. Turk J Gastroenterol.

[15] Wakeham K, Murray L, Muirhead R (2021). Multicentre investigation of prognostic factors incorporating p16 and tumour infiltrating lymphocytes for anal cancer after chemoradiotherapy. Clin Oncol (R Coll Radiol).

[16] Nigro ND, Seydel HG, Considine B (1983). Combined preoperative radiation and chemotherapy for squamous cell carcinoma of the anal canal. Cancer.

[17] Leichman L, Nigro N, Vaitkevicius VK (1985). Cancer of the anal canal. Model for preoperative adjuvant combined modality therapy. Am J Med.

[18] Tomaszewski JM, Link E, Leong T (2012). Twenty-five-year experience with radical chemoradiation for anal cancer. Int J Radiat Oncol Biol Phys.

[19] James RD, Glynne-Jones R, Meadows HM (2013). Mitomycin or cisplatin chemoradiation with or without maintenance chemotherapy for treatment of squamous-cell carcinoma of the anus (ACT II): a randomised, phase 3, open-label, 2 × 2 factorial trial. Lancet Oncol.

[20] Slørdahl KS, Klotz D, Olsen J (2021). Treatment outcomes and prognostic factors after chemoradiotherapy for anal cancer. Acta Oncol.

[21] Day FL, Link E, Ngan S (2011). FDG-PET metabolic response predicts outcomes in anal cancer managed with chemoradiotherapy. Br J Cancer.

[22] Flam M, John M, Pajak TF (1996). Role of mitomycin in combination with fluorouracil and radiotherapy, and of salvage chemoradiation in the definitive nonsurgical treatment of epidermoid carcinoma of the anal canal: results of a phase III randomized intergroup study. J Clin Oncol.

[23] Gunderson LL, Winter KA, Ajani JA (2012). Long-term update of US GI intergroup RTOG 98–11 phase III trial for anal carcinoma: survival, relapse, and colostomy failure with concurrent chemoradiation involving fluorouracil/mitomycin versus fluorouracil/cisplatin. J Clin Oncol.

[24] Leon O, Guren M, Hagberg O (2014). Anal carcinoma – survival and recurrence in a large cohort of patients treated according to Nordic guidelines. Radiother Oncol.

[25] Rusten E, Rekstad BL, Undseth C (2019). Anal cancer chemoradiotherapy outcome prediction using (18)F-fluorodeoxyglucose positron emission tomography and clinicopathological factors. Br J Radiol.

[26] Kochhar R, Renehan AG, Mullan D (2017). The assessment of local response using magnetic resonance imaging at 3- and 6-month post chemoradiotherapy in patients with anal cancer. Eur Radiol.

